# Protective Vaccination of Mice Against Blood-Stage Malaria Impacts Hepatic Expression of Genes Encoding Acute-Phase Proteins and IL-6 Family Members

**DOI:** 10.3390/ijms26073173

**Published:** 2025-03-29

**Authors:** Frank Wunderlich, Daniela Gerovska, Denis Delic, Marcos J. Araúzo-Bravo

**Affiliations:** 1 Department of Biology, Heinrich-Heine-University, 40225 Düsseldorf, Germany; frank.wunderlich@hhu-duesseldorf.de; 2 Computational Biology and Systems Biomedicine, Biogipuzkoa Health Research Institute, 20014 San Sebastian, Spain; danielaivanova.gerovska@bio-gipuzkoa.eus; 3 Boehringer Ingelheim Pharma & Co., KG, 88400 Biberach, Germany; 4 IKERBASQUE, Basque Foundation for Science, 48009 Bilbao, Spain; 5 Department of Cell Biology and Histology, Faculty of Medicine and Nursing, University of Basque Country (UPV/EHU), 48940 Leioa, Spain

**Keywords:** protective vaccination, blood-stage malaria, liver response, genes expressing plasma proteins, coagulation, fibrinolysis, complement, IL-6 trans-signaling

## Abstract

In response to vaccination and/or infectious agents, the liver produces acute-phase proteins (APPs) driven by IL-6, which circulate in blood plasma as components of the humoral innate defense. This study investigates the liver of mice for possible effects of protective vaccination against primary blood-stage infections of *Plasmodium chabaudi* malaria on the expression of genes encoding APPs and IL-6 family members. Female Balb/c mice were vaccinated with a non-infectious vaccine prior to challenge with 10^6^ *P. chabaudi-*infected erythrocytes, resulting in about 80% survival of otherwise lethal infections. Gene expression microarrays were used to determine the relative transcript levels of genes in the livers of vaccinated and unvaccinated mice on days 0, 1, 4, 8, and 11 *p.i.* (*post infectionem*). Vaccination induced significant (*p*-value < 0.05) differences in the expression of malaria-responsive genes toward the end of crisis on day 11 *p.i.*, when mice recovered from infections. These genes include *Saa4*, *Apcs*, *Cp*, and *Crp*, encoding APPs described to inhibitorily interact with parasitic blood stages; the genes *F2*, *F7*, *F8*, *F9*, *F10*, and *F13b*, and *Plg*, *Plat*, and *Serpina5*, encoding proteins balancing coagulation vs. fibrinolysis dysregulated by malaria, respectively; the genes *Hc*, *C8a*, *C8b*, *C8g*, and *C9*, encoding components of lytic complement membrane attack complex (MAC); and *Cfh*, *Cfi*, and *C4bp*, encoding complement-regulatory proteins. Vaccination accelerated, albeit differently, the malaria-induced activation of all three complement pathways, evidenced as higher transcript levels of *C1qa*, *C1qb*, *C1qc*, *Fcna*, *Cfp*, *C3*, *Cfh*, *C8a*, and *C9* on day 4 *p.i.*, *C1ra*, *C1s*, and *C2* on day 1 *p.i.*, and *Serping1*, encoding the multifunctional protease inhibitor C1INH, on day 0 *p.i.* Protective vaccination may also accelerate downregulation of the malaria-promoting lethality of IL-6 trans-signaling, which may contribute to an overall accelerated recovery of mice from otherwise lethal blood-stage malaria.

## 1. Introduction

Morbidity and mortality from malaria are caused by the blood stages of parasitic protozoans of the genus *Plasmodium*, which develop and multiply within host red blood cells. Worldwide, malaria was estimated to be responsible for 247 million cases and 619,000 deaths in 2021, with most deaths occurring in children under 5 years of age in Sub-Saharan African countries [1]. A commercial vaccine against malaria is not yet available, but promising progress has been made towards an effective anti-malaria vaccine [2,3]. Recently, the WHO recommended the RTS,S/AS01 vaccine, also known as Mosquirix, to be offered to young children aged 6 weeks to 17 months in African regions with moderate or high malaria transmission, but its efficacy in terms of reducing hospitalization is only about 30%, and wanes over time [1,4]. More recently, the presumably more effective R21/Matrix-M vaccine has been approved by the WHO for trials in young-aged children in four African countries [5,6,7].

More basic research is desirable to improve our understanding of the host defense mechanisms that need to be activated by vaccination to provide effective protection against blood-stage malaria. Such host mechanisms can be conveniently studied in animal models. One such experimental model is *P. chabaudi* in mice [8,9], which shares several characteristics with *P. falciparum*, the deadliest human malaria species, causing about 99% of malaria-related deaths worldwide. In *P. chabaudi* infections, an experimental non-infectious vaccine was shown to enable the curing of otherwise non-healing lethal blood-stage malaria [10,11]. This cure is associated with a reduced peak parasitaemia by about 30%, and changed responses of the liver to malaria. Thus, the liver of vaccinated mice responds to malaria with increased IFNγ production, attenuated inflammation, augmented uptake of injected particles [11], and changed expression of genes encoding not only mRNAs, but also miRNAs [12]. Importantly, protective vaccination accelerates malaria-induced liver-intrinsic extramedullary erythroblastosis [13], megakaryo-/thrombopoiesis [14], and NK cell generation [15]. These effects of vaccination support the view that the liver plays a potential role in the survival of otherwise lethal infections of blood-stage malaria [16].

The liver is also known for its ability to rapidly produce acute-phase proteins (APPs) in response to vaccinations and/or infectious agents, which play an effective role in the humoral innate host defense [17,18,19]. APPs circulate and patrol in the blood plasma, but are mainly produced by hepatocytes, which are the most abundant cell type in the liver and are immunological agents in their own right [20], and, to a lesser extent, by Kupffer cells, the resident macrophages of the liver. APPs exert manifold functions, and a given APP can even exert opposing functions [18]. There are positive and negative APPs, which are either up- or downregulated in response to infection. Negative APPs include albumin and transferrin, whereas most APPs are positive, such as C-reactive protein, serum amyloid A, caeruloplasmin, and haptoglobulin [18]. APP signatures are also used as diagnostic markers for diseases in human and veterinary medicine, and increasingly also for testing vaccine efficacy [17,21]. APPs are also critical for determining the course and outcome of blood-stage malaria. The quantity and quality of circulating APPs vary in patients infected with different *Plasmodium* species [22,23,24] and in different experimental blood-stage infections [16,25,26]. A recent meta-analysis even proposed the C-reactive protein as an early biomarker for malaria and for monitoring malaria severity [27]. Particularly critical in the context of outcomes of blood-stage malaria may also be those APPs that play central roles in blood coagulation and fibrinolysis, as well as in the complement (C) system.

The C system is a highly effective first line of the host humoral innate immunity against infectious diseases, including blood-stage malaria [28,29]. More than 60 fluid-phase and membrane-bound proteins act together against pathogens in tight, mainly proteolytically controlled sequential cleavage cascades and amplifications of different components [30]. There are three activation pathways: the classical pathway (CP), the lectin pathway (LP), and the alternative pathway (AP), which converge upon activation by pathogens at the level of C3 convertases, cleaving the APP C3—a key C component that promotes chemotaxis/vasodilatation, including attraction of macrophages and neutrophils and, in particular, opsonization of microbes as C3b. The C5b component initiates the formation of the membrane attack complex (MAC) with the final lytic C9 component [30]. However, there is also evidence that not only do malaria-infected hosts benefit from complement activation, but also, its dysregulation is increasingly being considered as causing pathogenesis in severe malaria [31]. Such dysregulations in complement activation may also critically affect blood coagulation and fibrinolysis, which are tightly intertwined with the C system [32,33]. For instance, fibrinogen as a central APP in blood coagulation is cleaved by thrombin to form fibrin and fibrin clots, respectively. The latter, in turn, can be dissolved with the participation of numerous protein factors, initiated by plasmin cleaved from the APP plasminogen. A well-regulated balance of coagulation and fibrinolysis appears to be required for a cure [34]. However, it remains poorly understood whether protective vaccination affects this regulation at all.

The main driver of APP production is the pleiotropic cytokine interleukin 6 (IL-6). IL-6 is produced, other than by immune cells, by hepatocytes in particular, which also express the specificity-defining membrane IL-6 receptor α (IL6Rα). Signal transduction occurs through the ubiquitously expressed IL-6 signal-transducing membrane receptor GP130 [35,36]. IL-6 is also known to play a critical role in the course and outcome of blood-stage malaria. Previously, malaria-induced lethality in mice infected with *P. chabaudi* has been shown to be overcome by interrupting the alternative IL-6 trans-signaling pathway mediated by IL-6 bound to the soluble (s)IL-6Rα [37]. The IL-6 family includes other members, such as IL-11, IL-27, IL-31, oncostatin M (OSM), leukemia inhibitory factor (LIF), ciliary neurotrophic factor (CTNF), cardiotrophin 1 (CTF-1), and cardiotrophin-like cytokine factor 1 (CLCF1) [35,36], which also use GP130 as a signal-transducing receptor, in part in combination with other receptors, such as LIF receptor (LIFR) and OSM receptor (OSMR). However, only a paucity of data are available on the impact of protective vaccination on the expression of the different IL-6 family members and their specificity-defining and signal-transducing receptors in the liver in response to blood-stage malaria.

The present study is a continuation of our previous studies on the role of the liver in vaccination-induced survival of blood-stage malaria: it aims to identify genes encoding blood plasma proteins and IL-6 family members, whose expression in the liver of mice is changed by protective vaccination against *P. chabaudi* blood-stage malaria. For this purpose, whole-genome oligo gene expression microarrays are a very appropriate tool to concomitantly compare the relative expression levels of many different genes at given times. Specifically, the same gene expression microarrays as used in our previous studies [13,14,15] will be used here for analysis of selected genes, whose expression in the liver responds to vaccination and to malaria in both vaccination-protected mice undergoing progressive blood-stage infection, and in unvaccinated mice suffering a lethal outcome. Vaccination-responsive genes in the liver may be of potential importance for vaccination-protected survival from the otherwise lethal outcome of primary blood-stage infections of *P. chabaudi* in unvaccinated mice.

## 2. Results

### 2.1. Responsiveness to Malaria and Vaccination of Hepatic Expression of Genes Encoding APPs

**Figure 1:**
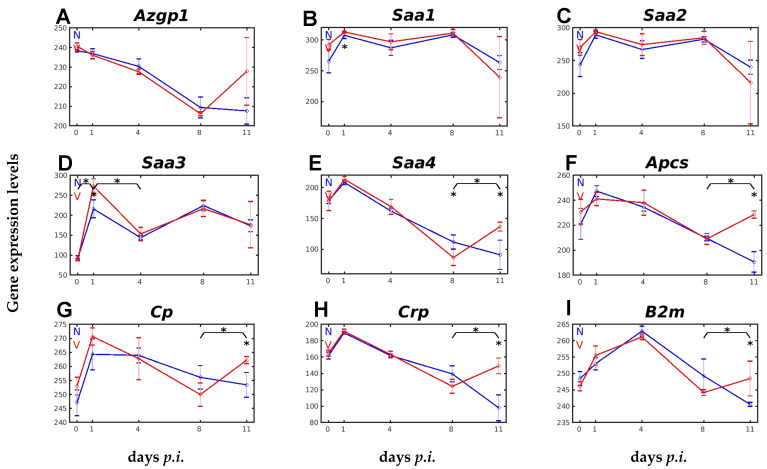
Expression trajectories of genes encoding different APPs in the liver in response to blood-stage malaria and vaccination. (**A**) *Azgp1*. (**B**) *Saa1*. (**C**) *Saa2*. (**D**) *Saa3*. (**E**) *Saa4*. (**F**) *Apcs*. (**G**) *Cp*. (**H**) *Crp*. (**I**) *B2m*. RNA was isolated from single livers of vaccination-protected (V, red) and non-vaccinated (N, blue) mice on days 0, 1, 4, 8, and 11 *p.i.* during primary infection with *P. chabaudi* blood-stage malaria. Gene expression levels are plotted on a linear scale as the mean of three microarrays +/− SD. The ‘*’ symbol over the sampling points or interval lines between two sampling points indicate a statistically significant difference, with a *p-*value < 0.05, at a given sampling point or two corresponding intervals between vaccinated and non-vaccinated mice. Numerical *p*-values are provided in Table S1. *n_N_* = 3 and *n_V_* = 3 are the total number of non-vaccinated and vaccinated mice, respectively, at each time point.

Figure 1 shows the expression patterns of malaria-responsive genes encoding APPs in the liver of female Balb/c mice, and their responsiveness to vaccination. The genes *Azgp1*, encoding α-2-glycoprotein1, and *Saa2*, encoding serum amyloid A2, were not responsive to vaccination, whereas the genes *Saa1*, *Saa3*, *Saa4*, *Apcs*, *Cp*, *Crp*, and *B2m*, encoding SAA1, SAA3, SAA4, serum amyloid P, Caeruloplasmin, C-reactive protein, and β-2 microglobulin, respectively, significantly responded to vaccination.

*Azgp1*, with a constitutive expression of about 240 above the normalization level, continuously decreased its expression to about 210 at the peak of parasitaemia on day 8 *p.i.*, and this level remained towards the end of the crisis phase of infection on day 11 *p.i.* (Figure 1A). Similarly, to unvaccinated mice, the hepatic expression of *Azgp1* in vaccinated mice took a decreasing course, with a minimum on day 8 *p.i.*, before displaying an insignificant increase towards the end of the crisis, on day 11 *p.i.* (Figure 1A). The expression of *Saa1* increased from about 263 to about 308 above normalization level in unvaccinated mice at early prepatency on day 1 *p.i.*, and this level of expression was maintained at early patency on day 4 *p.i.* and at peak parasitaemia on day 8 *p.i.*, before decreasing to about 263 towards the end of the crisis phase on day 11 *p.i.* (Figure 1B). *Saa2* showed a very similar expression profile to *Saa1*, though at slightly lower levels (Figure 1B,C). In contrast to *Saa1* and *Saa2*, the genes *Saa3* and *Saa4* displayed significantly lower constitutive expressions of about 90 and 178, respectively, and their malaria-responsive expression profiles responded to vaccination, though differently (Figure 1D,E). Upon infection, *Saa3* increased its initial expression to about 215 in the liver of unvaccinated mice and to 275 in vaccinated mice on day 1 *p.i.*, before both decreased to about 155 on day 4 *p.i.*, followed by identical expression towards the end of the crisis phase on day 11 *p.i*. *Saa4* also increased its initial expression from 178 to about 214 on day 1 *p.i.*, followed by a decrease in expression which reached a minimum on day 11 *p.i.* in unvaccinated mice, whereas this decrease reached its minimum on day 8 *p.i.* in vaccination-protected mice, before turning to a significant increase in expression on day 11 *p.i.* (Figure 1E). Similar expression patterns as seen for *Saa4*, i.e., a significant increase in expression towards the end of the crisis after a period of decrease in vaccinated mice compared to a further ongoing decrease in unvaccinated mice, were also observed for the genes *Apcs*, encoding the serum amyloid P (SAP) component, *Cp*, encoding caeruloplasmin, *Crp*, encoding C-reactive protein, and *B2m*, encoding β-2 microglobulin (Figure 1F–I).

Figure S1A–G shows the genes whose expression was responsive to malaria, but not to vaccination, which included *Hp*, coding for haptoglobin, *Orm1-3*, coding for the orosomucoids1-3, *Apoe*, coding for apolipoproteinE, *A2m*, encoding α-2-macroglobulin, and *Fn1*, encoding fibronectin1. These genes were constitutively expressed on day 0 *p.i.* at levels between 318 and 133 above normalization. Only *A2m* exhibited a very low constitutive level of expression (Figure S1F). The expression of these genes varied during the course of primary blood-stage infection. *Hp* and *Orm1-3* showed an increase in expression at early prepatency on day 1 *p.i.* (Figure S1A–D). The expression of *Apoe* and *A2m* remained largely unchanged until early patency on day 4 *p.i.*, before they began to respond significantly to malaria: *Apoe* with a decrease and *A2m* with an increase reaching a minimum and a maximum, respectively, at peak parasitaemia on day 8 *p.i.* (Figure S1E,F). In addition, *Fn1*, with a constitutive expression of about 254 above normalization, decreased its expression, upon malaria infection, to about 245 on day 4 *p.i.*, before increasing slightly again at peak parasitaemia (Figure S1G). Numerical *p*-values of Figure S1 are provided in Table S9.

The two genes—*Alb*, encoding the abundant APP albumin, and *Trf*, encoding transferrin—did not respond, neither to malaria nor to vaccination (Figure S1H,I). Albumin is critical for the regulation of oncotic pressure, acts as a carrier for various molecules, particularly fat-soluble molecules, and its concentration in the blood plasma is known to decrease in response to various infectious agents [17,18].

### 2.2. Vaccination Affects Expression of Genes Involved in Blood Coagulation and Fibrinolysis

**Figure 2:**
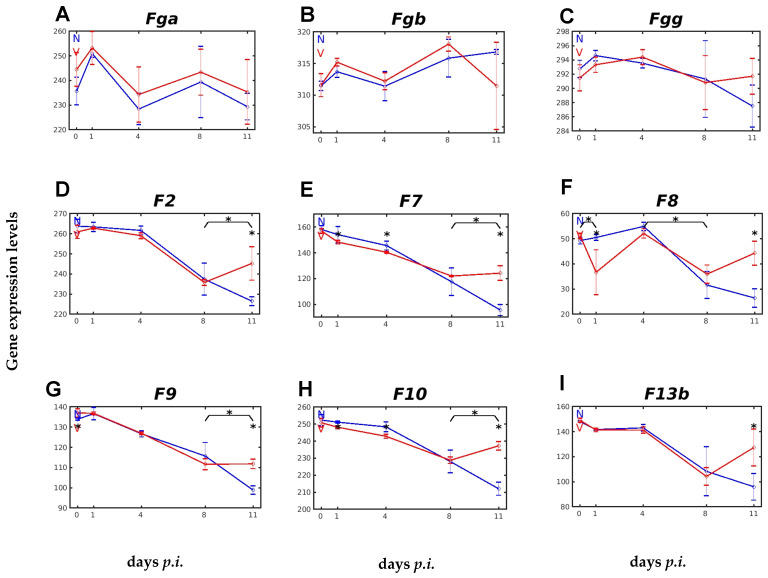
Time courses of genes encoding proteins involved in blood coagulation, expressed in the liver in response to malaria and vaccination. (**A**) *Fga*. (**B**) *Fgb*. (**C**) *Fgg*. (**D**) *F2*. (**E**) *F7*. (**F**) *F8*. (**G**) *F9*. (**H**) *F10*. (**I**) *F13b*. RNA was isolated from single livers of vaccination-protected (V, red) and non-vaccinated (N, blue) mice on days 0, 1, 4, 8, and 11 *p.i.* during primary infection with *P. chabaudi* blood-stage malaria. Gene expression levels are plotted on a linear scale as the mean of three microarrays +/− SD. The ‘*’ symbol over the sampling points or interval lines between two sampling points indicate a statistically significant difference, with a *p-*value < 0.05, at a given sampling point or two corresponding intervals between vaccinated and non-vaccinated mice. Numerical *p*-values are provided in Table S2. *n_N_* = 3 and *n_V_* = 3 are the total number of non-vaccinated and vaccinated mice, respectively, at each time point.

The soluble APP fibrinogen consists of the three subunits, α, β, and γ, which are encoded by the genes *Fga*, *Fgb*, and *Fgg*, whose expression was differentially responsive to malaria, but largely unresponsive to vaccination (Figure 2A–C). The fibrinogens FGA, FGB, and FGG are known to be cleaved by the serine protease thrombin, which is derived by proteolytic cleavage from the precursor prothrombin, to form the insoluble fibrin [38]. Expression of the prothrombin-encoding gene *F2* was responsive to malaria and vaccination (Figure 2D). This responsiveness was evidenced during crisis between days 8 and 11 *p.i.*, when *F2* expression in unvaccinated mice was further decreased towards the end of the crisis on day 11 *p.i.* Concomitantly, however, the decreasing *F2* expression in vaccination-protected mice at the peak of parasitaemia on day 8 *p.i.* was significantly reversed to increased expression (Figure 2D). Remarkably, such a significant turnaround in expression during crisis in vaccinated mice compared to unvaccinated mice could also be observed for the malaria-sensitive expression of the genes *F7*, *F8*, *F9*, *F10*, and *F13b*, which also encode proteins involved in the coagulation cascade (Figure 2E–I).

**Figure 3:**
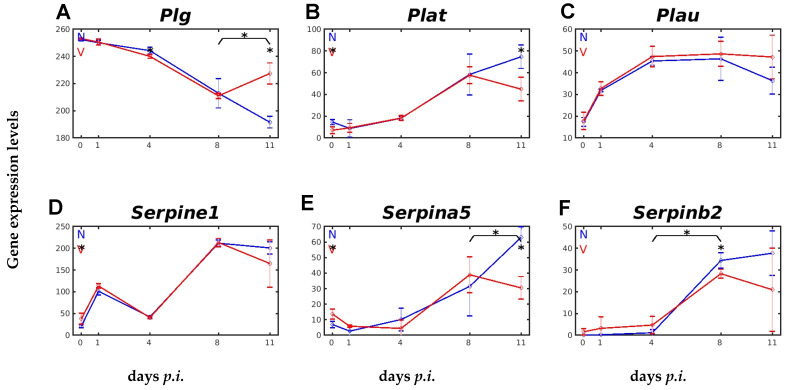
Hepatic expression of genes, encoding proteins involved in fibrinolysis, in response to malaria and vaccination. (**A**) *Plg*. (**B**) *Plat*. (**C**) *Plau*. (**D**) *Serpine1*. (**E**) *Serpina5*. (**F**) *Serpinb2*. RNA was isolated from single livers of vaccination-protected (V, red) and non-vaccinated (N, blue) mice on days 0, 1, 4, 8, and 11 *p.i.* during primary infection with *P. chabaudi* blood-stage malaria. Gene expression levels are plotted on a linear scale as the mean of three microarrays +/− SD. The ‘*’ symbol over the sampling points or interval lines between two sampling points indicate a statistically significant difference, with a *p-*value < 0.05, at a given sampling point or two corresponding intervals between vaccinated and non-vaccinated mice. Numerical *p*-values are provided in Table S3. *n_N_* = 3 and *n_V_* = 3 are the total number of non-vaccinated and vaccinated mice, respectively, at each time point.

Fibrinolysis, the disintegration of the insoluble fibrin clot, is then achieved mainly by the serine protease plasmin, which is proteolytically derived from the precursor plasminogen encoded by *Plg* [38]. The constitutive expression of *Plg* is slightly lower than that of *F2*, but the course of *Plg* expression in response to malaria and vaccination is very similar to that of *F2* (Figure 3A). In unvaccinated and vaccinated mice, there is a decrease in Plg expression until the peak of parasitaemia on day 8 *p.i.* A further decrease is followed in unvaccinated mice towards the end of the crisis at day 11 *p.i.*, whereas *Plg* expression is significantly increased during crisis in vaccinated mice (Figure 3A). A key enzyme in fibrinolysis is the serine protease PLAT (plasminogen activator, tissue type), which cleaves the proenzyme PLG to plasmin, which is thought to play a central role in orchestrating fibrinolysis, coagulation, and the complement system [32]. The gene *Plat*, encoding the plasminogen activator, which is tissue-type, showed a very low constitutive expression in both unvaccinated and vaccinated mice, and responded almost identically to malaria, with a relatively slow increase in expression, until peak parasitaemia was reached at day 8 *p.i.* During the crisis phase, however, *Plat* expression continued to increase in unvaccinated mice, reaching a maximum towards the end of the crisis at day 11 *p.i.*, whereas it decreased significantly at the same time in vaccinated mice (Figure 3B). Unlike *Plat*, the malaria-responsive expression of *Plau*, encoding the urokinase type plasminogen activator, was not vaccination-responsive (Figure 3C).

There exist three plasminogen activator inhibitors (PAIs): PAI-1, encoded by the gene *Serpine1*, PAI-2, encoded by *Serpinb2*, and PAI-3 (serine protease inhibitor, clade A, member 5), encoded by *Serpina5.* Protective vaccination induced a significant increase in the constitutive expression of *Serpine1* and *Serpina5* (Figure 3D,E). *Serpina5* displayed a significant response to vaccination, evidenced as decreasing expression during crisis in vaccinated mice when compared with more rapidly increasing expression in unvaccinated mice. The malaria-induced expression of *Serpinb2* showed a significantly faster increase in transcript levels between day 4 *p.i.* and day 8 *p.i.* in the liver of unvaccinated mice than in vaccinated mice, resulting in lower transcript levels at peak parasitaemia in vaccinated mice (Figure 3F).

### 2.3. Vaccination Impacts Malaria-Responsive Expression of Genes Encoding C Components

**Figure 4:**
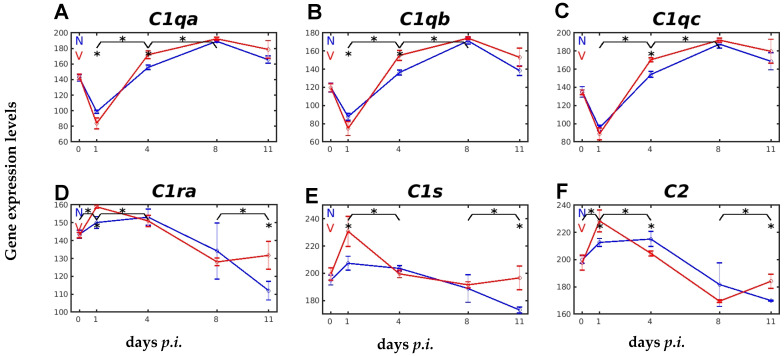
Expression of genes encoding early complement components of the CP in response to malaria and vaccination. (**A**) *C1qa*. (**B**) *C1qb*. (**C**) *C1qc*. (**D**) *C1ra*. (**E**) *C1s*. (**F**) *C2*. RNA was isolated from single livers of vaccination-protected (V, red) and non-vaccinated (N, blue) mice on days 0, 1, 4, 8, and 11 *p.i.* during primary infection with *P. chabaudi* blood-stage malaria. Gene expression levels are plotted on a linear scale as the mean of three microarrays +/− SD. The ‘*’ symbol over the sampling points or interval lines between two sampling points indicates a *p-*value < 0.05 statistically significant difference at a given sampling point or two corresponding intervals between vaccinated and non-vaccinated mice. Numerical *p*-values are provided in Table S4. *n_N_* = 3 and *n_V_* = 3 are the total number of non-vaccinated and vaccinated mice, respectively, at each time point.

Central to the C system is the APP C3, which is activated by all three activation pathways [30]. The first component of the CP, the C1 complex, circulates in the plasma and is composed of C1qr^2^s^2^ [39]. C1q consists of two A chains, two B chains, and two C chains, which are encoded by *C1qa*, *C1qb*, and *C1qc*, localized in a cluster on chromosome 4 [39]. In the liver of female Balb/c mice, these three genes were constitutively expressed at levels between about 120 and about 140 above the normalization level (Figure 4A–C). The courses of expression of these three *C1q* genes in response to malaria and vaccination were almost identical. Upon infection, there was a strong response of these transcript levels, characterized by a transient decrease on day 1 *p.i.*, which was followed by a massive increase on day 4 *p.i.*, reaching its maximum at the peak of parasitaemia on day 8 *p.i.* (Figure 4A–C). Remarkably, the transient decrease on day 1 *p.i.*, and especially the re-increase in expression on day 4 *p.i.*, were lower and higher, respectively, in vaccinated mice than in unvaccinated mice (Figure 4A–C). The expression of *C1ra* and C1s took a completely different pattern as compared with the *C1q* genes (Figure 4D,E). Indeed, infection of unvaccinated and vaccinated mice did not lead to transiently decreased mRNA levels of *C1ra* and *C1s* initially on day 1 *p.i.*, but instead, the levels increased. In vaccinated mice, this initial increase in C1ra and C1s mRNA levels was significantly accelerated, as well as there being a subsequent decline towards day 4 *p.i.*, before the decrease returned to elevated levels during the crisis phase (Figure 4D,E). An accelerated increase in expression on day 1 *p.i.* was also found for the *C2* gene in vaccinated mice, which was followed by an accelerated decreasing expression until peak parasitaemia on day 8 *p.i.*, as compared with unvaccinated mice. Towards the end of crisis on day 11 *p.i.*, the decreasing expression continued in unvaccinated mice, whereas expression in vaccinated mice increased (Figure 4F).

**Figure 5:**
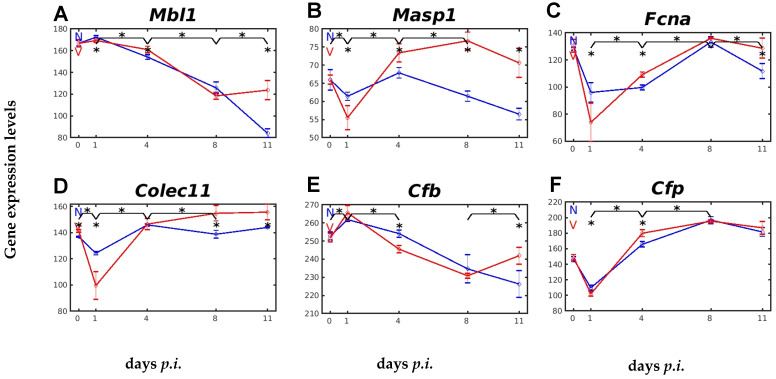
Expression trajectories of genes encoding components of the LP and AP in the liver of vaccinated and unvaccinated mice. (**A**) *Mbl1*. (**B**) *Masp1*. (**C**) *Fcna*. (**D**) *Colec11*. (**E**) *Cfb*. (**F**) *Cfp*. RNA was isolated from single livers of vaccination-protected (V, red) and non-vaccinated (N, blue) mice on days 0, 1, 4, 8, and 11 *p.i.* during primary infection with *P. chabaudi* blood-stage malaria. Gene expression levels are plotted on a linear scale as the mean of three microarrays +/− SD. The ‘*’ symbol over the sampling points or interval lines between two sampling points indicates a statistically significant difference, with a *p-*value < 0.05, at a given sampling point or two corresponding intervals between vaccinated and non-vaccinated mice. Numerical *p*-values are provided in Table S5. *n_N_* = 3 and *n_V_* = 3 are the total number of non-vaccinated and vaccinated mice, respectively, at each time point.

The LP is activated when pattern recognition molecules, such as mannose-binding lectin (MBL), other collectins, or ficolins bind to carbohydrates on the surface of pathogens [30]. However, the response of their encoding genes to malaria or vaccination is dramatically different. The expression of the genes *Mbl1*, encoding MBL1, *Masp1*, encoding the precursor MBL-associated serine protease1 (MASP1), *Fcna*, encoding ficulin A, and *Colec11*, encoding collectin 11, is shown in Figure 5A–D. Upon infection of unvaccinated mice, there was only a slight increase in *Mbl1* expression on day 1 *p.i.*, which was followed by an accelerating decrease in expression, reaching its lowest level towards the end of the crisis on day 11 *p.i.* Protective vaccination significantly changed the response to malaria of *Mbl* expression in the liver. There was no increase in expression on day 1 *p.i.*, and only a slightly slower decrease in expression until day 4 *p.i.* in vaccinated mice than in unvaccinated mice. After day 4 *p.i.*, *Mbl1* expression declined in both vaccinated and unvaccinated mice, but only until the peak of parasitaemia on day 8 *p.i.*, when the decline stopped and turned into an increased expression towards the end of the crisis on day 11 *p.i.* Unlike *Mbl1*, however, the expression of *Masp1* followed a different time course after infection, particularly in vaccinated mice, resembling more that of *C1qa*, *C1qb*, and *C1qc* (Figure 5B). There was initially a significantly transient decrease in its expression on day 1 *p.i.*, before the expression increased on day 4 *p.i.* and reached a maximum on day 8 *p.i.*, which was higher than the corresponding expression level in unvaccinated mice (Figure 5B).

Remarkably, the expression trajectories of *Fcna* and *Colec11* in the liver of vaccinated mice infected with *P. chabaudi* took a similar course to that of *Masp1*, with an initial strong transient decrease on day 1 *p.i.*, followed by a faster increase at day 4 *p.i.*, peaking on day 8 *p.i.* (Figure 5C,D). It is also notable that the vaccination response of *Colec11* differed from that of both *Fcna* and *Masp1*, insofar as vaccination per se increased the constitutive expression of *Colec11*, and the initial transient decrease in the malaria-responsive expression was much lower in the liver of vaccinated mice than in unvaccinated mice (Figure 5B–D).

The constitutively active AP also appeared to respond to malaria and vaccination, as evidenced by the expression profiles of complement factors B and P (properdin) [30]. The gene *Cfb*, encoding factor B, had a much higher constitutive expression than *Cfp*, encoding properdin. Upon infection, there was an initial increase in expression that peaked at early prepatency on day 1 *p.i.*, followed by a decline in expression that was faster in vaccinated mice than in unvaccinated mice, i.e., *Cfb* transcript levels were lower in vaccinated mice than in unvaccinated mice on day 4 *p.i.* (Figure 5E). At the peak of parasitaemia on day 8 *p.i.*, expression continued to decrease in unvaccinated mice during crisis, whereas Cfb transcript levels in vaccinated mice increased significantly at the same time (Figure 5E). The gene *Cfp* displayed a very similar expression pattern as *C1qa*, *C1qb*, and *C1qc* in response to blood-stage malaria, with a faster increase towards day 4 *p.i.* after an initial faster decrease on day 1 *p.i.* in vaccinated mice than in unvaccinated mice (Figure 5F). The same maximum expression was reached in both vaccinated and unvaccinated mice at peak parasitaemia on day 8 *p.i.*

**Figure 6:**
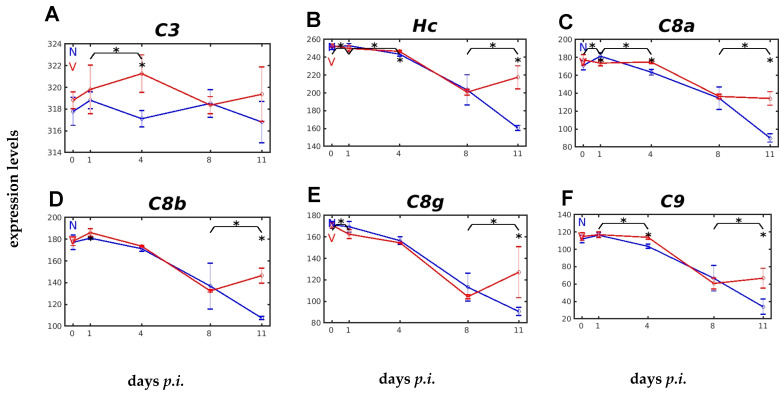
Time courses of hepatic expression of genes encoding C3, C5, and final components of the membrane attack complex (MAC). (**A**) *C3*. (**B**) *Hc*. (**C**) *C8a*. (**D**) *C8b*. (**E**) *C8g*. (**F**) *C9*. RNA was isolated from single livers of vaccination-protected (V, red) and non-vaccinated (N, blue) mice on days 0, 1, 4, 8, and 11 *p.i.* during primary infection with *P. chabaudi* blood-stage malaria. Gene expression levels are plotted on a linear scale as the mean of three microarrays +/− SD. The ‘*’ symbol over the sampling points or interval lines between two sampling points indicates a statistically significant difference, with a *p-*value < 0.05, at a given sampling point or two corresponding intervals between vaccinated and non-vaccinated mice. Numerical *p*-values are provided in Table S6. *n_N_* = 3 and *n_V_* = 3 are the total number of non-vaccinated and vaccinated mice, respectively, at each time point.

All three complement pathways converge at the level of C3 convertases, incidentally via C4 and C2 in the CP and LP pathways [30]. The gene encoding C3 showed a relatively high constitutive expression of about 318 above normalization level in both vaccinated and unvaccinated mice (Figure 6A). Upon infection of unvaccinated mice, the *C3* expression levels remained largely unaffected, whereas an increase in expression occurred in vaccinated mice, with a maximum at early patency on day 4 *p.i.* (Figure 6A).

The final membrane attack complex (MAC) is initiated by activation of C5, encoded by *Hc*, which binds C6, C7, C8, and the highly lytic C9 [30,40,41]. Figure 6B–F show the expression of the *Hc*, *C8a*, *C8b*, and *C9* genes. Conspicuously, the expression of these genes was similar in response to blood-stage malaria, but with significant differences between vaccination-protected and unvaccinated mice. In unvaccinated mice, there was occasionally a slight increase in expression on day 1 *p.i.*, before it began to decrease to its lowest level towards the end of the crisis on day 11 *p.i.* (Figure 6B–F). However, the expression of *C8a* and *C9* in vaccinated mice was significantly higher on day 4 *p.i.* than in unvaccinated mice. Thereafter, expression declined towards day 8 *p.i.*, but became reversed significantly during crisis (Figure 6C–F).

### 2.4. Vaccination Impacts Expression of Genes Encoding Complement Regulatory Proteins

**Figure 7:**
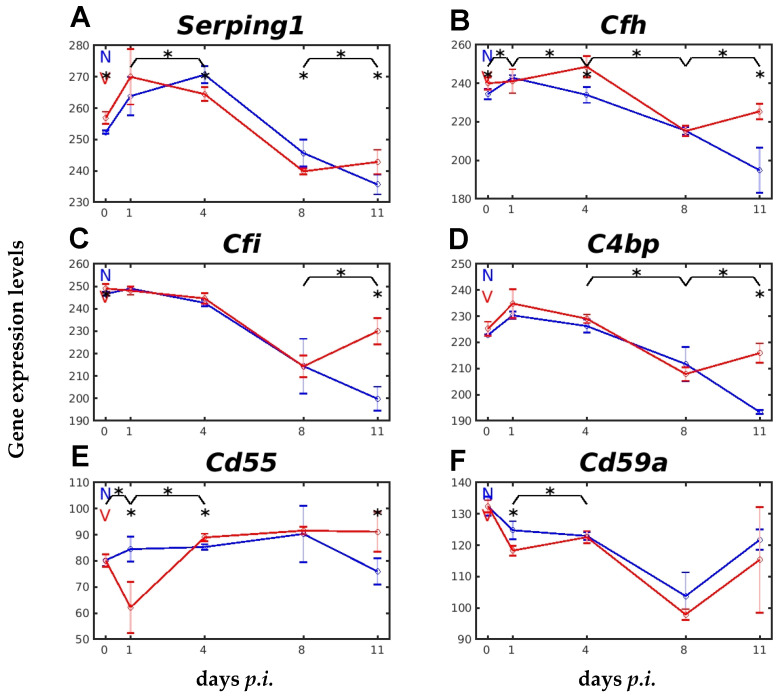
Expression of genes encoding different complement regulatory proteins in vaccination-protected and unvaccinated mice infected with *P. chabaudi* malaria. (**A**) *Serping1*. (**B**) *Cfh*. (**C**) *Cfi*. (**D**) *C4bp*. (**E**) *Cd55*. (**F**) *Cd59a*. RNA was isolated from individual livers taken from vaccination-protected (V, red) and non-vaccinated (N, blue) mice on days 0, 1, 4, 8, and 11 *p.i.* during primary infections with *P. chabaudi* blood-stage malaria. Gene expression levels are plotted on a linear scale as the mean of three microarrays +/− SD. The ‘*’ symbol over the sampling points or interval lines between two sampling points indicate a statistically significant difference, with a *p-*value < 0.05, at a given sampling point or two corresponding intervals between vaccinated and non-vaccinated mice. Numerical *p*-values are provided in Table S7. *n_N_* = 3 and *n_V_* = 3 are the total number of non-vaccinated and vaccinated mice, respectively, at each time point.

The liver is also known to express specific C regulatory proteins to prevent overactivation of various C components [42]. For instance, the C inhibitor (C1INH), encoded by *Serping1* (serine or cysteine peptidase inhibitor, clade G, member 1), is known to inactivate the C1 complex in the CP and the MASP-1 and MASP-2 in the LP. The *Cfh-*encoded Factor H, the *Cfi*-encoded Factor I, the *C4bp-*encoded C4BP, and the *Cd55-*encoded CD55 are known to be negative regulators of AP, and the membrane-localized CD59 encoded by *Cd59* inhibits the MAC [30,38,42]. Notably, the expression of these genes was both malaria- and vaccination-responsive, but in different ways (Figure 7A–F). For instance, vaccination per se significantly increased the constitutive expression of *Serping1* and *Cfh*. Furthermore, the initial increase in *Serping1* expression in the liver of vaccinated mice on day 1 *p.i.* correlated with the initially transient decrease in expression of *C1q* genes (Figure 4A–C) and *Masp1* (Figure 5B), as well as with that of *Cd55* and Cd59a (Figure 7E,F). The expression of *Serping1*, *Cfh*, *Cfi*, *C4bp*, and *CD55* at day 11 *p.i.* was significantly greater in vaccinated mice than in unvaccinated mice (Figure 7A–E).

### 2.5. Vaccination Affects Hepatic Gene Expression of IL-6 Family Members in Response to Blood-Stage Malaria

**Figure 8:**
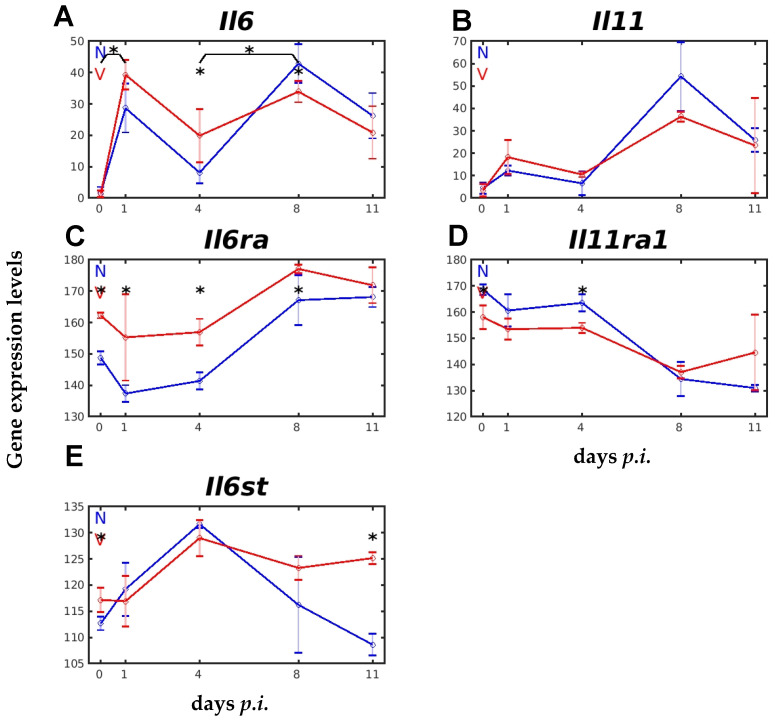
Expression trajectories of *Il6*, *Il11*, and corresponding receptors in the liver of vaccination-protected and unvaccinated mice during primary infection with *P. chabaudi* blood-stage malaria. (**A**) *Il6*. (**B**) *Il11*. (**C**) *Il6ra*. (**D**) *Il11ra1*. (**E**) *Il6st*. RNA was isolated from individual livers of vaccination-protected (V, red) and non-vaccinated (N, blue) mice on days 0, 1, 4, 8, and 11 *p.i.* during primary infection with *P. chabaudi* blood-stage malaria. Gene expression levels are plotted on a linear scale as the mean of three microarrays +/− SD. The ‘*’ symbol over the sampling points or interval lines between two sampling points indicates a statistically significant difference, with a *p-*value < 0.05, at a given sampling point or two corresponding intervals between vaccinated and non-vaccinated mice. Numerical *p*-values are provided in Table S8. *n_N_* = 3 and *n_V_* = 3 are the total number of non-vaccinated and vaccinated mice, respectively, at each time point.

Figure 8 shows the time course of expression of *Il6*, *Il6ra*, and *Il6st* in the liver of unvaccinated and vaccinated mice in response to primary blood-stage infection with *P. chabaudi* malaria. All three genes were constitutively expressed on day 0 *p.i.*, but at different levels. *Il6* was expressed at a very low level of about 4 above normalization level, *Il6ra* at the highest level of about 149, and *Il6st* at about 113, in unvaccinated mice. All three genes were responsive to blood-stage malaria (Figure 8A–C). Upon infection of unvaccinated mice, the initial expression of *Il6* on day 1 *p.i*. was upregulated, like that of *Il6st*, whereas that of *Il6ra* was significantly downregulated. The expression of *Il6* in the liver of unvaccinated mice was significantly downregulated on day 4 *p.i.*, before being upregulated again on day 8 *p.i.*, and then declining on day 11 *p.i.* to approximately the level observed on day 1 *p.i. IL6ra* expression on day 4 *p.i.* remained at about the same reduced level observed on day 1 *p.i.*, but then increased, reaching its maximum at peak of parasitaemia on day 8 *p.i.*, which was maintained towards the end of the crisis phase on day 11 *p.i.* (Figure 8B). Protective vaccination did not affect the low constitutive expression level of *Il6*, but significantly affected that of *Il6st* and that of *Il6ra*. Protective vaccination also affected the course of the malaria-responsive expression of these genes. The initial increase in *IL6* expression during early prepatency was faster, the decreased expression on day 4 *p.i.* was higher, the increase between day 4 and 8 *p.i.* was slower, and the second peak of expression was lower in vaccinated mice than in unvaccinated mice (Figure 8A). The course of *Il6ra* expression in vaccinated mice was similar to that in unvaccinated mice, but the relative transcript levels were significantly greater in vaccinated mice on days 1, 4, and 8 *p.i.*, possibly due to the increased constitutive expression induced by vaccination (Figure 8B). Also, the constitutive expression of *Il6st* was significantly greater in the liver of vaccinated mice than in unvaccinated mice (Figure 8C). Upon infection, the *Il6st* expression in unvaccinated mice was highly elevated, peaking at day 4 *p.i.*, before decreasing steadily towards the end of the crisis phase at day 11 *p.i*. In contrast to unvaccinated mice, the *Il6st* expression in vaccination-protected mice was delayed on day 1 *p.i.*, before reaching its maximum on day 4 *p.i.*, and then remained at this high level until day 11 *p.i.*, i.e., the *Il6st* expression was significantly greater in vaccination-protected mice than in unvaccinated mice during crisis (Figure 8C).

Besides IL-6, IL-11 is the only member of the IL6 family which uses two recruited GP130 signal transducers [36]. In response to blood-stage malaria, *Il11* expression took a two-peak course that was not significantly different between unvaccinated and vaccinated mice. The first peak at day 1 *p.i.* was much lower than the second peak at day 8 *p.i.* (Figure 8D). By contrast, the expression of *Il11ra1* encoding the IL-11 specificity-defining receptor was highly responsive to vaccination. Its constitutive expression was reduced on day 0 *p.i.*, and this reduced expression level remained reduced on day 4 *p.i.* in the liver of vaccinated mice compared to unvaccinated mice (Figure 8E).

Il27 signals through the membrane receptor IL27Rα, in association with one GP130 [43]. The genes *Il27* and *Il27ra* were constitutively expressed in the liver, and their expressions were malaria-responsive, but only *Il27ra* was also significantly responsive to vaccination (Figure S2A,B). The malaria-responsive expression of *Il27ra* was initially decreased on day 1 *p.i.*, then increased on day 4 *p.i.*, and this expression level was maintained until day 11 *p.i.* in vaccination-protected mice compared to unvaccinated mice (Figure S2B). Numerical *p*-values of Figure S2 are provided in Table S10.

OSM also uses a single GP130 in combination with either OSMR or LIFR. The GP130/LIFR heterodimer is also used by LIF and CTF1 [36]. The expression of *Osm*, *Lif*, *Ctf1*, and *Lifr* were sensitive to both malaria and vaccination. In particular, vaccination per se decreased the constitutive expression of *Ctf1*, while the expression of *Osm* and *Lif* was initially increased on day 1 *p.i.*, and the expression of *Lifr* was increased towards the end of the crisis (Figure S3A–E). Numerical *p*-values of Figure S3 are provided in Table S11. LIFR/GP130 can also be used by CRLF1, CLCF1, and CNTF, with CNTFR as the specificity-defining membrane receptor [36]. The expression of *Ctnfr* was decreased on day 4 *p.i.*, and that of *Ctnf* on day 11 *p.i.*, in the liver of vaccinated mice compared to unvaccinated mice (Figure S4A–D). Numerical *p*-values of Figure S4 are provided in Table S12.

## 3. Discussion

Our data reveal significant differences in the expression of genes in the liver of vaccination-protected vs. unvaccinated mice during primary blood-stage infection with *P. chabaudi* malaria. Such differences were most evident late in infection, i.e., during the crisis phase between peak parasitaemia on day 8 *p.i.* and dramatically falling parasitaemias towards the end of the crisis on day 11 *p.i.* Higher expression levels were identified in vaccination-protected mice for the genes *Cp*, *Saa4*, *Apcs*, *B2m*, and *Crp*; for the genes *F2*, *F7*, *F8*, *F9*, *F10*, and *F13b*, encoding proteins involved in the coagulation cascade; for the gene *Plg*, encoding the zymogen of plasmin involved in fibrinolysis; and mostly for those genes encoding various complement components involved in the activation and regulation of complements such as *C1ra*, *C1s*, *Mbl1*, *Masp1*, *Colec11*, *Fcna*, *Cfb*, *C2*, *Hc*, *C8a*, *C8b*, *C8g*, *C9*, *Serping1*, *Cfh*, *Cfi*, *C4bp*, and *Cd55*. Conspicuously, these increased transcript levels occur during crisis, i.e., the infection phase when the liver has resumed increased activity, which was previously evidenced as an increased uptake by the liver of fluorescent particles injected into vaccination-protected infected mice [11]. It is therefore reasonable to assume that these genes encode APPs, which may necessarily contribute to processes required for the overall recovery of the mice from infection during the crisis phase.

This assumption is more plausible for genes encoding those APPs known to inhibitorily interact with *Plasmodium*-infected erythrocytes and/or merozoites. For example, our data are consistent with previous results showing that the serum of mice at the beginning of the crisis phase of self-healing *P. chabaudi* malaria infections contained elevated levels of the *Cp*-encoded caeruloplasmin, which binds copper known for its contact killing properties [25]. The short pentameric pentraxin SAP encoded by *Apcs* is able to inhibit the growth of the intraerythrocytic malaria parasites [44]. Another member of the pentraxin family, namely CRP, is also known to bind to *Plasmodium*-infected erythrocytes, presumably via host Fcγ receptors [45,46]. CRP is a planar ring-shaped molecule consisting of five non-covalently linked protomers with an A- and a B-face [47]. It is the ligand-binding B-face that is capable of opsonizing microbial pathogens, including *Plasmodium*-infected erythrocytes and presumably merozoites. Such binding may explain why ascending parasitaemia in *P. chabaudi* infections, from early patency on day 4 *p.i.* to peak parasitaemia on day 8 *p.i.*, is associated with decreasing levels of Crp transcripts. The A-face of CRP is known to interact with C1q through its globular head region [48]. Binding of CRP to *Plasmodium*-infected erythrocytes activates complement, leading to hemolysis and erythrocyte clearance [45]. Consistent with this, the increased Crp mRNA production in the liver of vaccination-protected mice during crisis runs in parallel with concomitantly increased hepatic mRNA production of the genes *Hc*, *C8a*, *C8c*, and *C9*, encoding components of the terminal C cascade. This suggests concomitant actions of CRP and the lytic MAC on the parasite blood stages, i.e., *P. chabaudi*-parasitized erythrocytes and free merozoites, during the crisis phase of infection. Concomitantly with increased MAC formation during crisis, the liver produces increasing mRNA levels of *Cfh*, *Cfi*, *C4bp*, and *Cd55*, encoding complement regulatory proteins protecting host cells from lysis, e.g., non-infected erythrocytes and thrombocytes [33]. It is attractive to speculate that particularly reticulocytes, which may make up about 90% of all red blood cells during crisis [11], may benefit from these complement regulatory proteins, since reticulocytes are not the favorite host cells of *P. chabaudi* merozoites [13].

Moreover, a late response to malaria in the liver of vaccinated mice was also found for the expression of genes encoding plasma proteins involved in coagulation and fibrinolysis. Although the malaria-responsive expression of the genes *Fga*, *Fgb*, and *Fgg*, encoding the subunits of the APP fibrinogen, was not vaccination-responsive, the transcript levels of genes involved in the coagulation cascade, such as *F2*, encoding the zymogen prothrombin, *F7*, *F8*, *F9*, *F10*, and *F13b*, were highly significantly increased in the liver of vaccinated mice during the crisis, whereas, concomitantly, the expression of these genes continued to decrease in unvaccinated mice. Moreover, the hepatic expression of genes involved in fibrinolysis also showed a late vaccination response. The expression of *Plg*, encoding the APP plasminogen, was increased during the crisis only in vaccinated mice, even at slightly higher levels than the vaccination-responsive *F* genes (Figure 3A). A key enzyme in fibrinolysis is the serine protease PLAT (plasminogen activator, tissue type), which cleaves the proenzyme PLG to plasmin, which, in turn, plays a central role in orchestrating fibrinolysis, coagulation, and the contact and complement system [32,49]. Consistently with this, *Plat* expression was also found to respond to vaccination late in the crisis phase. Unlike *Plg*, however, *Plat* mRNA levels did not further increase, but decreased during crisis. In addition, the decreasing *Plat* mRNA levels during crisis were found to run in parallel with decreased expression of *Serpina5*, encoding the plasminogen activator inhibitor PAI-3, in the liver of vaccinated mice during the crisis. This suggests a delicately balanced regulation, not only at the mRNA level, between PLG, on the one hand, and PLAT and PAI-3, on the other hand, during the crisis phase of infections: decreasing levels of PAI-3 and PLAT during crisis in vaccination-protected mice could explain the re-increase in PLG levels. Reversely, increased expression of *Plat* and *Serpina5* and, concomitantly, decreased expression of *Plg* during crisis, as observed in unvaccinated mice, may have resulted in an increased dysregulation of the ‘fibrinolytic activity’ in unvaccinated mice. Possibly, this may have ultimately led to their death, due to increased formation of thrombi, which may have still been promoted by the increased generation of platelets in the liver, as previously found during crisis [14]. Evidence that PAIs may be critical for regulating blood-stage malaria was previously found in *P. chabaudi*-selfhealer C57BL/6 mice: these mice, when depleted of *Serpine1* encoding PAI-1, partially lost their ability to control the course of infection of *P. chabaudi* malaria [50].

Furthermore, our data indicate that protective vaccination impacts, albeit differently, the activation of the three malaria-responsive complement pathways AP, LP, and CP. Even within a given pathway, vaccination differently impacts the individual complement factors. For instance, the constitutively active AP is characterized by spontaneously hydrolysing C3 and its initial association with Factor B, which is cleaved by Factor D, generating the alternative C3 convertase, which, in turn, is stabilized by the positive AP regulator properdin and is negatively regulated by Factor H [30]. Consistently with this, our data show, at the mRNA level, that protective vaccination accelerates the downregulation of initially upregulated Cfb transcripts in the liver on day 4 *p.i.*, but, concomitantly, accelerates an upregulation Cfh and Cfp transcripts (Figure 5E,F and Figure 7B). Furthermore, our data show the responses to malaria of both the CP and the LP to be also accelerated by vaccination. The genes *C1qa*, *C1qb*, and *C1qc*, encoding the subunits of the circulating C1q complex in the CP, had significantly higher expression at early patency on day 4 *p.i.* (Figure 4A–C), just as the genes encoding *Mbl1*, *Masp1*, and *Fcna* in the LP (Figure 5A–C). However, the accelerated LP response to vaccination on day 4 *p.i.* is complex, as the MBL1-mediated pathway appears to be sequentially implemented by an accelerated ficolin-mediated pathway and by a collectin11-mediated pathway, evidenced by the accelerated increase in mRNA levels of *Fcna* at day 4 *p.i.* and *Colec11* at peak parasitaemia on day 8 *p.i.* The surface binding activity of ficolin and collectin11 appears to be associated with increasing parasitaemias in the peripheral blood [11]. Both the LP and CP, as well as the AP, eventually converged at the level of C3-convertase, and the C3 mRNA level also increased more rapidly in the liver of vaccinated mice on day 4 *p.i.* than in unvaccinated mice, i.e., vaccination accelerated the formation of C3, induced by primary infection with *P. chabaudi* blood-stage malaria, and its cleavage product C3b is known to opsonize malaria-infected erythrocytes and merozoites, respectively [51].

The view that vaccination accelerates activation of the CP and LP is further substantiated with the higher levels of *C1ra*, *C1s*, *C2*, and *Serping1* in vaccination-protected mice than in unvaccinated mice at early prepatency on day 1 *p.i.* (Figure 4D–F and Figure 7A). Even the transiently decreased levels of *C1qa*, *C1qb*, *C1qc*, *Masp1*, *Fcna*, and *Colec11* on day 1 *p.i.* in the liver of vaccinated mice appeared to be more lowered than in unvaccinated mice. These transiently decreased levels may reflect an apparent rapid consumption of these transcripts in the early activation patterns of both the CP and the LP. For example, the initiation complex of the CP is an assembly of the recognition protein C1q with two proteases C1r and two proteases C1s, the latter being responsible for the activation and proteolytic activity of the C1 complex [30,39]. Conspicuously, our data show that the mRNA level of *C1ra*, which encodes the subcomponent A of C1r, is highly significantly increased by vaccination at early prepatency on day 1 *p.i.*, in contrast to its impaired increase in unvaccinated mice (cf. Figure 4). Furthermore, active C1r, in turn, is known to cleave, and, thus, to activate C1s, which then splits C4 and C2 to produce C4a, C4b, C2a, and C2b, and C4b combines with C2a to generate C3- or C5-convertase [30]. In accordance, this may also explain why an accelerated increase in the expression of *C1s* and *C2* was found in vaccinated mice on day 1 *p.i.*, in contrast to the impaired lower increases in C1s and C2 mRNAs in unvaccinated mice (cf. Figure 4). The initially increased mRNA expression of *C1ra*, *C1s*, and *C2* in the liver of vaccinated mice suggests that protective vaccination accelerates the complex malaria-induced initial activation patterns of CP and LP.

A dominant regulatory role in the complex control of complement activation, but also in other non-complement-mediated actions, may be ascribed to the C1INH encoded by *Serping1*, a fluid-phase multifunctional serine protease inhibitor [30,52,53]. The C1INH is known to limit overactivation of the complement system, thereby protecting host tissues from aberrant C activation. C1INH negatively regulates the CP by binding to the C1q-complex, and the LP by inhibiting MASP1 and MASP2 [52,54]. C1INH also interacts with coagulation and fibrinolysis by binding to and inhibiting the serine proteases FXIIa, plasmin, and PLAT [52]. In addition, C1INH was described to suppress malaria parasite invasion of host erythrocytes and cytoadhesion by binding to parasite glycosylphosphatidylinositol and the host cell receptors CD36 and chondroitin sulfate A [55]. Recruitment of C1INH to *P. falciparum* malaria merozoites, in turn, controls complement activation [54]. Our data show that transcript levels of *Serping1* respond directly to vaccination: (i) vaccination per se increased the constitutive transcript levels of *Serping1*; (ii) upon infection with *P. chabaudi* malaria, *Serping1* mRNA increased more rapidly, with an early peak on day 1 *p.i.*, in vaccinated mice, whereas the peak in unvaccinated mice was delayed on day 4 *p.i.*; (iii) the decline in *Serping1* mRNA levels after the peak on day 1 *p.i.* towards day 8 *p.i.* was accelerated in vaccinated mice, before increasing again towards the end of the crisis, due to overall recovery from infection. The early increase in *Serping1* mRNA levels, and the subsequent accelerated decline, may have helped to limit the initial overactivation of the CP and LP by transiently impairing the initial activation components of the LP and CP on day 1 *p.i.*, characterized by the transient decrease in mRNA levels of *Masp1* and *C1q*, respectively. The ensuing accelerated downregulation of *Serping1* may, in turn, reflect that less C1INH is required, or may even be a hindrance, during this period of infection, whereas C1INH is required again for the final recovery from infection. Our data support the view that protective vaccination presumably accelerates activation and production of complement components in the liver, but concomitantly prevents overactivation of the complement system in response to malaria blood stages. It is tempting to speculate that such an accelerated production may facilitate a somewhat earlier formation of protective anti-malaria antibodies during the crisis phase, ultimately favoring survival from otherwise lethal *P. chabaudi* blood-stage infections.

Furthermore, our data show that protective vaccination also affects, albeit differently, the hepatic mRNA levels of some members of the IL-6 family, in particular, those of their specificity-defining and signal-transducing receptors. Among the IL-6 family members, significant changes (*p*-value < 0.05) were found for *Ctf1*, whose constitutive mRNA level was reduced by vaccination per se, and remained lower upon malaria infection compared to in unvaccinated mice, but only for the first 4 days of infection (cf. Figure S3). This downregulation might be not explainable by previous findings showing that CTF1 can induce an acute-phase response in rat hepatocytes [56], but may possibly be related to the pro- and/or anti-inflammatory properties of CTF1 [57,58]. Moreover, vaccination highly significantly increased *Lif* mRNA levels, but only at early prepatency on day 1 *p.i.* (cf. Figure S3). This appears to be consistent with previous data showing that LIF is able to induce an acute-phase response [56,59,60,61] and cachexia [62]. In contrast to *Lif* and *Ctf1*, vaccination had only a moderate effect (*p*-value < 0.05) on the malaria-induced IL-6 mRNA levels in the liver on day 1 *p.i.* Surprisingly, however, vaccination induced a significant increase in the constitutive mRNA level of Il6ra, encoding the membrane-bound IL6Rα, which remained elevated until the peak of parasitaemia on day 8 *p.i.* (cf. Figure 8). Vaccination did not affect IL-11 mRNA levels, but downregulated the constitutive mRNA levels of *Il11ra1*, which remained downregulated during the first 4 days of infection. This may be explained by limiting hepatoxic effects of IL-11 [63].

Abundant information, allowing a more reasonable interpretation, is only available for IL-6 and its receptors. Indeed, the mRNA levels of *Il6st*, encoding the IL-6 signal-transducing receptor GP130, were found to be upregulated by vaccination: at the constitutive level and during recovery from infection towards the end of the crisis at day 11 *p.i.* (cf. Figure 8). This suggests a critical role for GP130 in the recovery of vaccination-protected mice from *P. chabaudi* infections. Notably, the shed, soluble (s)GP130 has previously been described to function as a natural inhibitor of IL-6 trans-signaling [64], i.e., the sGP130 is able to bind and inactivate the soluble complex of IL-6/sIL6Rα, responsible for IL-6 trans-signaling [65]. In accordance, our previous study demonstrated that inhibition of IL-6 trans-signaling protects against malaria-induced lethality in mice [37]. The present results can therefore be interpreted as suggesting that protective vaccination may promote an accelerated downregulation of IL-6 trans-signaling, thereby favoring the resumption of processes in the liver required for accelerated recovery from infection during the crisis phase and, thus, for the survival of mice from otherwise lethal *P. chabaudi* blood-stage malaria.

Collectively, our data further substantiate the view that the liver plays an important role in the host defense against blood-stage malaria, and that protective vaccination may even enhance the liver’s responsiveness to experimental blood-stage infections of *P. chabaudi* malaria, evidenced as significant effects on constitutive and infection-altered expression of genes encoding various APPs and IL-6 family members.

## 4. Material and Methods

### 4.1. Blood-Stage Malaria of P. chabaudi

Infection experiments were previously performed with female Balb/c mice aged 10–12 weeks, bred under specified pathogen-free conditions, using a non-clonal line of *P. chabaudi* with similar features to *P. chabaudi chabaudi* AS [11,12,13]. The outcome of these blood-stage infections of malaria was previously shown to be controlled by genes of the H-2-complex and the non-H-2 background, and by sex and testosterone [66].

### 4.2. Protective Vaccination

Vaccination experiments were previously performed using a non-infectious vaccine consisting of erythrocyte membranes isolated as ghosts from *P. chabaudi*-parasitized erythrocytes by a glycerol-enhanced hypotonic shock, as detailed previously [11,13]. These membrane ghosts were previously shown to contain parasite-synthesized proteins, and presumably autoantigens [67,68]. Approximately 10^6^ ghosts suspended in 100 µL Freund’s complete adjuvant (FCA) were subcutaneously injected at weeks 3 and 1 *a.i.* (*ante infectionem*), before challenging with 10^6^ *P. chabaudi*-parasitized erythrocytes. These infections in vaccinated mice (V) took a similar course in terms of parasitaemia as those in non-vaccinated mice (N). In both V and N, the patent period began at about 4 *p.i.* with about 1–5% *P. chabaudi*-parasitized erythrocytes. Then, parasitaemia increased and culminated at peak parasitaemia on day 8 *p.i.*, with about 60% in V and about 40% in N [12]. The following crisis phase lasted for about 3–5 days, with parasitaemia descending to 5–2% at day 11 *p.i.*, and there was death of all N and survival of about 75% V [12], which corresponds with our previous results [11]. The efficacy of vaccination depends on sex and testosterone, respectively, and genes of the H-2 complex and non-H-2 background, as briefly summarized elsewhere [14,69].

### 4.3. Collection of Livers and RNA Preparation

Three V mice and three N mice were sacrificed at different time points during infections, i.e., on day 0 *p.i.*, at early prepatency on day 1 *p.i.*, at early patency on day 4 *p.i.*, at peak parasitaemia on day 8 *p.i*., and towards the end of the crisis phase on day 11 *p.i.* Livers were excised from the 30 sacrificed mice, rapidly frozen in liquid nitrogen, and stored at -80^o^ C until use. Total RNA was isolated from individual livers using the standard Trizol protocol (Qiagen, Hilden, Germany) followed by purification with the miRNeasy Kit (Qiagen) [13]. The integrity and quality of RNA was checked by the Agilent 2100 bioanalyzer platform (Agilent Technologies, Santa Clara, CA, USA), with RIN values of all 30 RNA samples being in the range between 8.7 and 9.1 [13].

### 4.4. Hybridization and Analyses of Mouse Whole-Genome Oligo Microarrays

These experiments have also previously been performed [13]. Cy3-labeled cRNA from 100 ng RNA equivalents of the 30 individual livers were generated using the Agilent Low Input Quick Amp Labeling Kit (Agilent Technologies). The incorporations ranged between 18 and 23 fmol Cy3/ng cRNA, as determined with an ND-spectrophotometer (NanoDrop Technologies, Wilmington, DE, USA). The Agilent Gene Expression Hybridization Kit was used to hybridize the Cy3-labeled cRNA to the Agilent’s 8x60K oligo microarrays (design number 028005), displaying 39,430 Entrez Gene RNAs. The Agilent’s Microarray Scanner System (Agilent Technologies) with the Agilent Feature Extraction Software (FES) 11.0 were used to read out and process the microarray image files. FES determines feature intensities including background subtraction, rejects outliers, and calculates the statistical confidence of the array spots. Using the quantile method, the microarrays were normalized across all 30 samples. Both raw and normalized data were made accessible in both the EMBL-EBI Array Express repository (array accession number: E-MTAB-4791) and the NCBI’s Gene Expression Omnibus (GEO) database with accession number GSE129133. All 30 normalized microarrays were previously subjected to global transcriptomic analyses, i.e., principal component analysis, a heat map of the most highly variable transcripts, and hierarchical clustering dendrograms calculated using the unweighted pair group method with arithmetic mean and Euclidean distance measure [13]. The study here analyzes the normalized microarrays with respect to the expression of selected genes encoding acute-phase proteins, including proteins involved in coagulation, fibrinolysis, and complement activation, as well as proteins encoding IL-6 family members and their receptors, respectively. The light intensities of single spots above the normalization level defined the relative levels of gene expression, which were represented as the mean ± SD as a dispersion metric in all figures.

### 4.5. Statistical Analysis

The non-parametric Krustall–Wallis test [70], conducted with the kruskalwallis function of Matlab (R2020b) (MathWorks, Inc. Natick, MA, USA), was used to determine the statistical significance of differences in the expression levels of a given gene between vaccinated mice (V) and non-vaccinated (N) mice on a given sampling day (0, 1, 4, 8, and 11 *p.i*), i.e., the means obtained from the three microarrays prepared from three individual livers of V (*n_V_* = 3) and from the three corresponding N microarrays (*n_V_* = 3) were compared. This test was also used to compare the statistical differences between the means for V and those for N over the intervals between different consecutive sampling days *p.i*. In all figures, statistical significance for a given sampling time or corresponding intervals between sampling times for vaccinated and non-vaccinated mice, with a *p*-value below the significance level α = 0.05, is indicated by an asterisk (*). The exact numerical *p*-values are provided in the Supplementary Tables corresponding to the figures. In these tables, the ’T’ columns show the *p*-values from the Kruskal–Wallis test for statistical significance of the difference at a given time point between the two groups, while the ‘Δ’ columns show the *p*-values from the Kruskal–Wallis test for the difference between two time points.

## 5. Limitations

Future studies are needed to confirm the findings regarding the protein level at selected time points in a larger number of animals. Freund’s adjuvant is considered highly immunostimulatory, and adding an adjuvant-only group would further enhance the vaccination-specific effects. Functional studies, such as targeting the complement pathway, are needed to uncover causative effects derived from the vaccination-induced protective effects.

## Data Availability

Microarray data are available at the NCBI’s GEO database with accession number GSE129133.

## Supplementary Materials

The following supporting information can be downloaded at https://www.mdpi.com/article/10.3390/ijms26073173/s1.
