# Supplementary material for: Protective Vaccination of Mice Against Blood-Stage Malaria Impacts Hepatic Expression of Genes Encoding Acute-Phase Proteins and IL-6 Family Members
Source: Int J Mol Sci. 2025 Mar 29;26(7):3173. doi: 10.3390/ijms26073173 (PMC11989154; doi:10.3390/ijms26073173)
Supplement: Supplementary file 1 [file ijms-26-03173-s001.zip › ijms-3508991-supplementary.pdf]

Supplementary Material

Protective Vaccination of Mice Against Blood-Stage Malaria Impacts Hepatic Expression of Genes Encoding Acute Phase Proteins and IL-6 Family Members

Frank Wunderlich [1,†], Daniela Gerovska [2,†], Denis Delic [3,*] and Marcos J. Araúzo-Bravo [2,4,5,*]

[1] Department of Biology, Heinrich-Heine-University, 40225 Düsseldorf, Germany; frank.wunderlich@hhu-duesseldorf.de
[2] Computational Biology and Systems Biomedicine, Biogipuzkoa Health Research Institute, 20014 San Sebastian, Spain; danielaivanova.gerovska@bio-gipuzkoa.eus
[3] Boehringer Ingelheim Pharma & Co. KG, 88400 Biberach, Germany
[4] IKERBASQUE, Basque Foundation for Science, 48009 Bilbao, Spain
[5] Department of Cell Biology and Histology, Faculty of Medicine and Nursing, University of Basque Country (UPV/EHU), 48940 Leioa, Spain
* Correspondence: delic@boeringer-ingelheim.com (D.D.); mararabra@yahoo.co.uk (M.J.A.-B.)
† These authors contributed equally to this work.

Table S1. Related to Figure 1. Columns T have the *p*-values of the Kruskal-Wallis test of the statistical significance of the difference in instant T of the two series. Columns Δ have the *p*-values of the Kruskal-Wallis test of the statistical significance of the difference between two time points.

Gene	T_0	T_1	T_4	T_8	T_{11}	Δ_{0-1}	Δ_{1-4}	Δ_{4-8}	Δ_{8-11}
Cp	0.1266	0.8273	0.0495	0.2752	0.0495	0.5127	0.1266	0.1266	0.0495
Saa3	0.2752	0.0495	0.5127	0.2752	0.5127	0.0495	0.0495	0.5127	0.8273
Saa1	0.1266	0.0495	0.2752	0.6579	0.5127	0.1266	0.5127	0.5127	0.5127
Saa2	0.1266	0.2752	0.2752	0.8273	0.5127	0.1266	0.8273	0.8273	0.5127
B2m	0.1266	0.2752	0.1266	0.1266	0.0495	0.1266	0.2752	0.2752	0.0495
Apcs	0.2752	0.1266	0.8273	0.8273	0.0495	0.1266	0.1266	0.2752	0.0495
Crp	0.2752	0.2752	0.5127	0.1266	0.0495	0.5127	0.5127	0.1266	0.0495
Saa4	0.8273	0.1266	0.5127	0.0495	0.0495	0.5127	0.8273	0.1266	0.0495
Azgp1	0.1266	0.2752	0.2752	0.2752	0.2752	0.1266	0.8273	0.8273	0.1266

Table S2. Related to Figure 2. Columns T have the *p*-values of the Kruskal-Wallis test of the statistical significance of the difference in instant T of the two series. Columns Δ have the *p*-values of the Kruskal-Wallis test of the statistical significance of the difference between two time points.

Gene	T_0	T_1	T_4	T_8	T_{11}	Δ_{0-1}	Δ_{1-4}	Δ_{4-8}	Δ_{8-11}
Fgb	0.8248	0.0765	0.6579	0.3758	0.5066	0.2752	0.5127	0.5127	0.1266
Fga	0.1266	0.8273	0.2752	0.5127	0.8273	0.2752	0.5127	0.8273	0.8273
Fgg	0.3758	0.1046	0.2683	1.0000	0.1775	1.0000	0.1266	0.8273	0.2752
F9	0.0495	0.5127	0.8273	0.2752	0.0495	0.1266	0.5127	0.5127	0.0495
F7	0.5127	0.0463	0.0495	0.5127	0.0495	0.1266	0.8273	0.5127	0.0495
F10	0.2752	0.0495	0.0495	0.8273	0.0495	0.5127	0.2752	0.5127	0.0495
F2	0.1212	0.8273	0.1840	0.5127	0.0495	0.5127	0.2752	0.8273	0.0495
F13b	0.5127	0.5127	0.8273	0.8273	0.0495	0.1266	0.8273	0.8273	0.1266
F8	0.1266	0.0495	0.1266	0.5127	0.0495	0.0495	0.1266	0.0495	0.1266

Table S3. Related to Figure 3. Columns T have the *p*-values of the Kruskal-Wallis test of the statistical significance of the difference in instant T of the two series. Columns Δ have the *p*-values of the Kruskal-Wallis test of the statistical significance of the difference between two time points.

Gene	T_0	T_1	T_4	T_8	T_{11}	Δ_{0-1}	Δ_{1-4}	Δ_{4-8}	Δ_{8-11}
Plg	0.2752	0.8248	0.0495	0.5127	0.0495	0.8273	0.1266	0.5127	0.0495
Plau	0.8273	0.5127	0.5127	0.8273	0.1266	0.8273	0.5127	0.8273	0.2752
Serpine1	0.0495	0.1266	0.8273	0.8273	0.2752	0.8273	0.1266	0.8273	0.2752
Serpinb2	0.5127	0.8273	0.2752	0.0495	0.2752	0.8273	0.8273	0.0495	0.5127
Plat	0.0495	0.8273	0.8273	0.8273	0.0495	0.2752	0.8273	0.8273	0.1266
Serpina5	0.0495	0.5127	0.2752	0.5127	0.0495	0.1266	0.1266	0.2752	0.0495

Table S4. Related to Figure 4. Columns T have the *p*-values of the Kruskal-Wallis test of the statistical significance of the difference in instant T of the two series. Columns Δ have the *p*-values of the Kruskal-Wallis test of the statistical significance of the difference between two time points.

Gene	T_0	T_1	T_4	T_8	T_{11}	Δ_{0-1}	Δ_{1-4}	Δ_{4-8}	Δ_{8-11}
C1qc	0.8273	0.1266	0.0495	0.2752	0.2752	0.2752	0.0495	0.0495	0.2752
C1qa	0.5127	0.0495	0.0495	0.1266	0.1266	0.1266	0.0495	0.0495	0.2752
C1qb	0.8273	0.0495	0.0495	0.1266	0.1266	0.2752	0.0495	0.0495	0.1266
C1ra	0.8273	0.0495	0.8273	0.5127	0.0495	0.0495	0.0495	0.5127	0.0495
C2	0.8273	0.0495	0.0463	0.1266	0.0495	0.0495	0.0495	0.5127	0.0495
C1s	0.2752	0.0495	0.2752	0.8273	0.0495	0.1266	0.0495	0.5127	0.0495

Table S5. Related to Figure 5. Columns T have the *p*-values of the Kruskal-Wallis test of the statistical significance of the difference in instant T of the two series. Columns Δ have the *p*-values of the Kruskal-Wallis test of the statistical significance of the difference between two time points.

Gene	T_0	T_1	T_4	T_8	T_{11}	Δ_{0-1}	Δ_{1-4}	Δ_{4-8}	Δ_{8-11}
Cfb	0.5127	0.1266	0.0495	0.5127	0.0495	0.0495	0.0495	0.5127	0.0495
Mbl1	0.5127	0.0495	0.0495	0.1266	0.0495	0.5127	0.0495	0.0495	0.0495
Masp1	0.8273	0.0495	0.0495	0.0495	0.0495	0.0495	0.0495	0.0495	0.5127
Colec11	0.0495	0.0495	0.8273	0.0495	0.0495	0.0495	0.0495	0.0495	0.5127
Cfp	0.5127	0.0495	0.0495	0.8273	0.2752	0.1266	0.0495	0.0495	0.2752
Fcna	0.5127	0.0495	0.0495	0.5127	0.0495	0.1266	0.0495	0.0495	0.0495

Table S6. Related to Figure 6. Columns T have the *p*-values of the Kruskal-Wallis test of the statistical significance of the difference in instant T of the two series. Columns Δ have the *p*-values of the Kruskal-Wallis test of the statistical significance of the difference between two time points.

Gene	T_0	T_1	T_4	T_8	T_{11}	Δ_{0-1}	Δ_{1-4}	Δ_{4-8}	Δ_{8-11}
C3	0.2386	0.6374	0.0463	0.8137	0.1967	0.8166	0.0463	0.0722	0.3758
C8g	0.8273	0.1266	0.5127	0.2752	0.0495	0.0495	0.2752	0.8273	0.0495
C8b	0.8273	0.0495	0.2752	0.5127	0.0495	0.5127	0.5127	0.5127	0.0495
Hc	0.5127	0.2752	0.0495	0.5127	0.0495	0.0495	0.0495	0.5127	0.0495
C9	0.2752	0.8273	0.0495	0.8273	0.0495	0.2752	0.0495	0.1266	0.0495
C8a	0.1266	0.0495	0.0495	0.8273	0.0495	0.0495	0.0495	0.2752	0.0495

Table S7. Related to Figure 7. Columns T have the *p*-values of the Kruskal-Wallis test of the statistical significance of the difference in instant T of the two series. Columns Δ have the *p*-values of the Kruskal-Wallis test of the statistical significance of the difference between two time points.

Gene	T_0	T_1	T_4	T_8	T_{11}	Δ_{0-1}	Δ_{1-4}	Δ_{4-8}	Δ_{8-11}
Cd59a	0.8273	0.0495	0.8273	0.5127	0.5127	0.1266	0.0495	0.5127	0.8273
Serping1	0.0495	0.2752	0.0495	0.0495	0.0495	0.8273	0.0495	0.8273	0.0495
C4bp	0.5127	0.5127	0.2752	0.3758	0.0495	0.2752	0.2752	0.0495	0.0495
Cfh	0.0495	0.8273	0.0495	0.8273	0.0495	0.0495	0.0495	0.0495	0.0495
Cfi	0.0495	0.5127	0.2752	0.8273	0.0495	0.1266	0.5127	0.8273	0.0495
Cd55	0.5127	0.0495	0.0495	0.5127	0.0495	0.0495	0.0495	0.5127	0.2752

Table S8. Related to Figure 8. Columns T have the *p*-values of the Kruskal-Wallis test of the statistical significance of the difference in instant T of the two series. Columns Δ have the *p*-values of the Kruskal-Wallis test of the statistical significance of the difference between two time points.

**Gene**	T_0	T_1	T_4	T_8	T_{11}	Δ_{0-1}	Δ_{1-4}	Δ_{4-8}	Δ_{8-11}
Il6	0.8273	0.1266	0.0495	0.0495	0.2752	0.0495	0.5127	0.0495	0.8273
Il11	0.8273	0.2752	0.1266	0.1266	0.5127	0.5127	0.8273	0.1266	0.2752
Il6ra	0.0495	0.0495	0.0495	0.0495	0.5127	0.8273	0.5127	0.2752	0.8273
Il6st	0.0495	0.5127	0.2752	0.5127	0.0495	0.1266	0.8273	0.1266	0.1266
Il11ra1	0.0495	0.1266	0.0495	0.8273	0.2752	0.5127	0.2752	0.1266	0.2752

Table S9. Related to Figure S1. Columns T have the *p*-values of the Kruskal-Wallis test of the statistical significance of the difference in instant T of the two series. Columns Δ have the *p*-values of the Kruskal-Wallis test of the statistical significance of the difference between two time points.

**Gene**	T_0	T_1	T_4	T_8	T_{11}	Δ_{0-1}	Δ_{1-4}	Δ_{4-8}	Δ_{8-11}
Alb	0.3173	0.3173	0.1213	NaN	0.4561	0.1967	1.0000	0.1213	0.4561
Trf	1.0000	0.8222	0.3758	0.5127	0.2463	0.6579	0.8273	0.5127	0.2752
A2m	0.8273	0.1266	0.8273	0.5127	0.8273	0.1266	0.1266	0.5127	0.1266
Fn1	0.1266	0.1840	0.2752	0.5127	0.1840	0.8273	0.5127	0.5127	0.2752
Hp	0.6579	0.3758	1.0000	0.3758	0.4867	0.5127	0.2752	0.6579	0.8273
Orm1	0.5127	0.5127	0.8273	0.8273	0.5127	0.5127	0.8273	0.5127	0.5127
Orm2	0.8273	0.5127	0.2752	0.8273	0.8273	0.5127	0.2752	0.5127	0.5127
Orm3	0.8273	0.5127	0.5127	0.5127	0.5127	0.5127	0.5127	0.5127	0.5127
Apoe	0.1266	0.1840	0.1266	0.8248	0.8273	0.6579	0.5127	0.2752	0.8273

Table S10. Related to Figure S2. Columns T have the *p*-values of the Kruskal-Wallis test of the statistical significance of the difference in instant T of the two series. Columns Δ have the *p*-values of the Kruskal-Wallis test of the statistical significance of the difference between two time points.

Gene	T_0	T_1	T_4	T_8	T_{11}	Δ_{0-1}	Δ_{1-4}	Δ_{4-8}	Δ_{8-11}
Il27	0.2752	0.8273	0.8273	0.5127	0.2752	0.8273	0.5127	0.8273	0.2752
Il27ra	0.2752	0.0495	0.2752	0.8273	0.0495	0.0495	0.0495	0.5127	0.0495

Table S11. Related to Figure S3. Columns T have the *p*-values of the Kruskal-Wallis test of the statistical significance of the difference in instant T of the two series. Columns Δ have the *p*-values of the Kruskal-Wallis test of the statistical significance of the difference between two time points.

Gene	T_0	T_1	T_4	T_8	T_{11}	Δ_{0-1}	Δ_{1-4}	Δ_{4-8}	Δ_{8-11}
Osm	0.5127	0.0495	0.0495	0.5127	0.2752	0.0495	0.5127	0.5127	0.5127
Osmr	0.2752	0.1266	0.5127	0.5127	0.5127	0.2752	0.5127	0.5127	0.5127
Lif	0.2752	0.0495	0.2752	0.5127	0.1266	0.0495	0.0495	0.1266	0.1266
Lifr	0.2752	0.5127	0.5127	0.0495	0.0495	0.2752	0.2752	0.1266	0.1266
Ctf1	0.0495	0.0495	0.0495	0.1266	0.8273	0.2752	0.2752	0.2752	0.8273

Table S12. Related to Figure S4. Columns T have the *p*-values of the Kruskal-Wallis test of the statistical significance of the difference in instant T of the two series. Columns Δ have the *p*-values of the Kruskal-Wallis test of the statistical significance of the difference between two time points.

**Gene**	T_0	T_1	T_4	T_8	T_{11}	Δ_{0-1}	Δ_{1-4}	Δ_{4-8}	Δ_{8-11}
Crlf1	0.8273	0.5127	0.2752	0.5127	0.5127	0.2752	0.5127	0.2752	0.5127
Cntf	0.5127	0.1266	0.1266	0.5127	0.0495	0.1266	0.5127	0.8273	0.0495
Cntfr	0.2752	0.5127	0.0495	0.5127	0.5127	0.2752	0.0495	0.2752	0.8273
Clcf1	0.8273	0.5127	0.5127	0.5127	0.2752	0.5127	0.8273	0.5127	0.1266

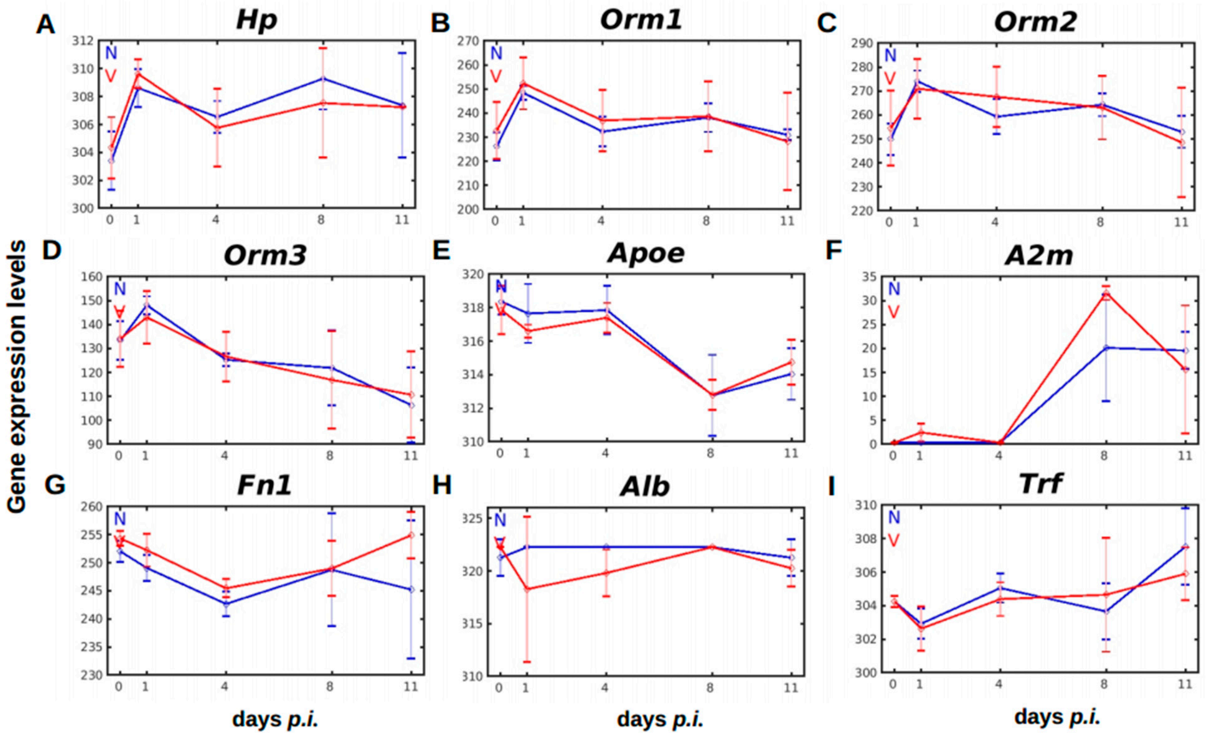

Figure S1. Expression trajectories of genes encoding different APPs in the liver in response to blood-stage malaria and vaccination. (A) *Hp*. (B) *Orm1*. (C) *Orm2*. (D) *Orm3*. (E) *Apoe*. (F) *A2m*. (G) *Fn1*. (H) *Alb*. (I) *Trf*. RNA was isolated from individual livers taken from vaccination-protected (V, red) and non-vaccinated (N, blue) mice on days 0, 1, 4, 8, and 11 *p.i.* during primary infections with *P. chabaudi* blood-stage malaria. Gene expression levels are plotted in linear scale as the mean of three microarrays +/- SD. The '*' symbol over the sampling points or interval lines between two sampling points indicates a *p*-value<0.05 statistically significant difference at a given sampling point or two corresponding intervals between vaccinated and non-vaccinated mice. Numerical *p*-values are provided in Table S9. n_N = 3 and n_V = 3 are the total number of non-vaccinated and vaccinated mice, respectively, at each time point. In contrast to *Alb* and *Trf*, the expression of *Hp*, *Orm1-3*, *Apoe*, *A2m*, and *Fn1* is malaria-responsive, but unresponsive to vaccination.

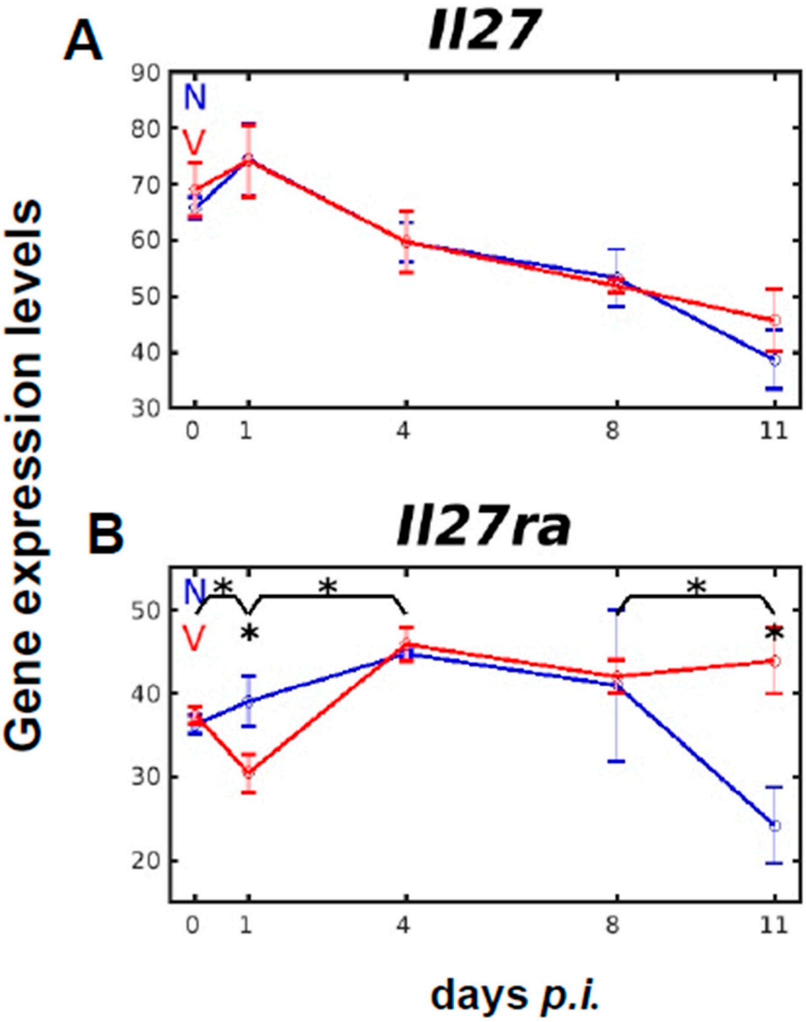

Figure S2. Expression of *Il27* (A) and *Il27ra* (B) in response to malaria and vaccination in the liver of vaccination-protected and unvaccinated mice. RNA was isolated from individual livers taken from vaccination-protected (V, red) and non-vaccinated (N, blue) mice on days 0, 1, 4, 8, and 11 *p.i.* during primary infections with *P. chabaudi* blood-stage malaria. Gene expression levels are plotted in linear scale as the mean of three microarrays +/- SD. The '*' symbol over the sampling points or interval lines between two sampling points indicates a p-value<0.05 statistically significant difference at a given sampling point or two corresponding intervals between vaccinated and non-vaccinated mice. Numerical p-values are provided in Table S10. $n_N = 3$ and $n_V = 3$ are the total number of non-vaccinated and vaccinated mice, respectively, at each time point.

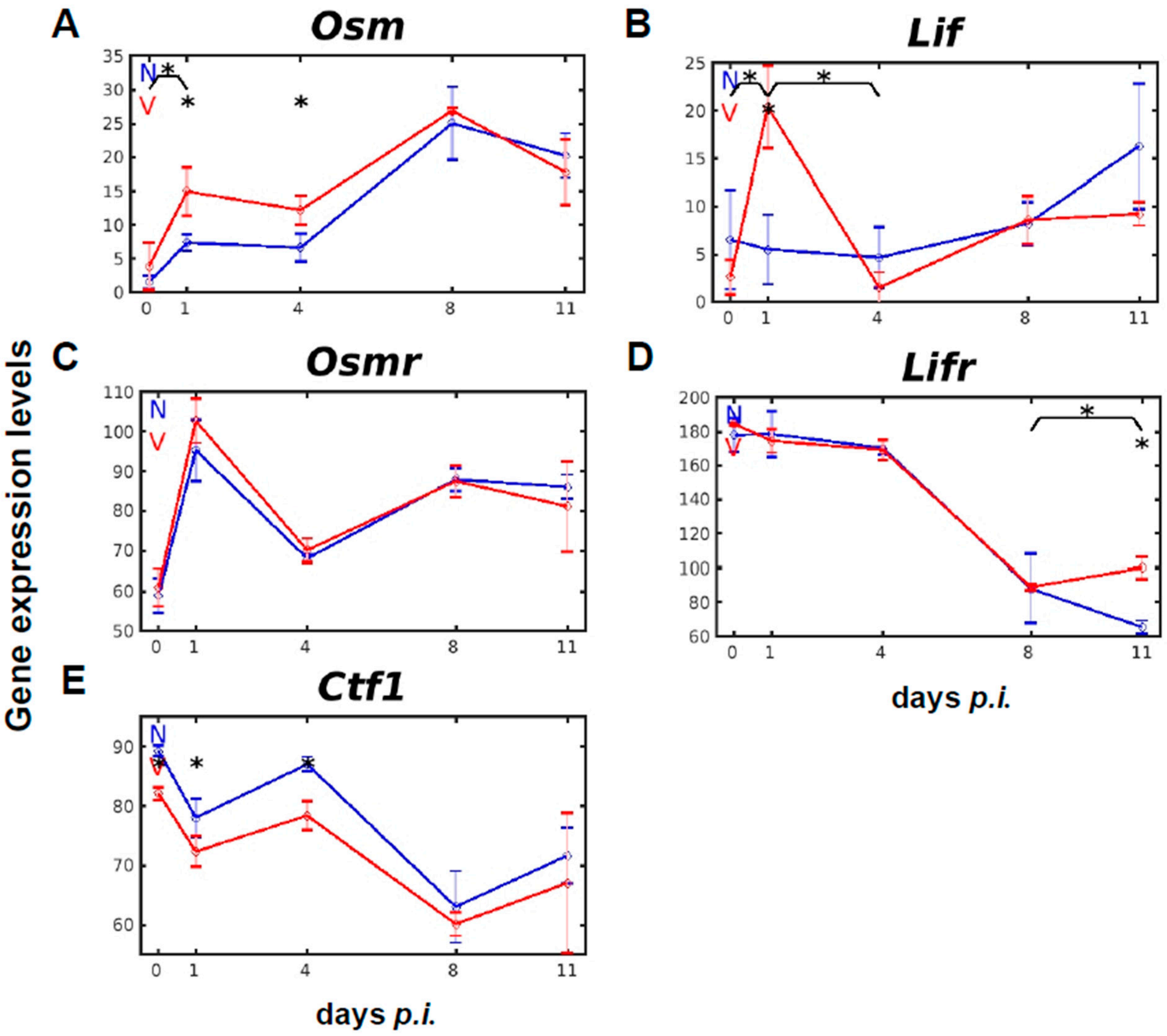

Figure S3. Expression trajectories of genes encoding different APPs in the liver in response to blood-stage malaria and vaccination. (A) *Osm*. (B) *Lif*. (C) *Osmr*. (D) *Lifr*. (E) *Ctf1*. RNA was isolated from individual livers taken from vaccination-protected (V, red) and non-vaccinated (N, blue) mice on days 0, 1, 4, 8, and 11 *p.i.* during primary infections with *P. chabaudi* blood-stage malaria. Gene expression levels are plotted in linear scale as the mean of three microarrays +/- SD. The '*' symbol over the sampling points or interval lines between two sampling points indicates a p-value<0.05 statistically significant difference at a given sampling point or two corresponding intervals between vaccinated and non-vaccinated mice. Numerical p-values are provided in Table S11. $n_N = 3$ and $n_V = 3$ are the total number of non-vaccinated and vaccinated mice, respectively, at each time point.

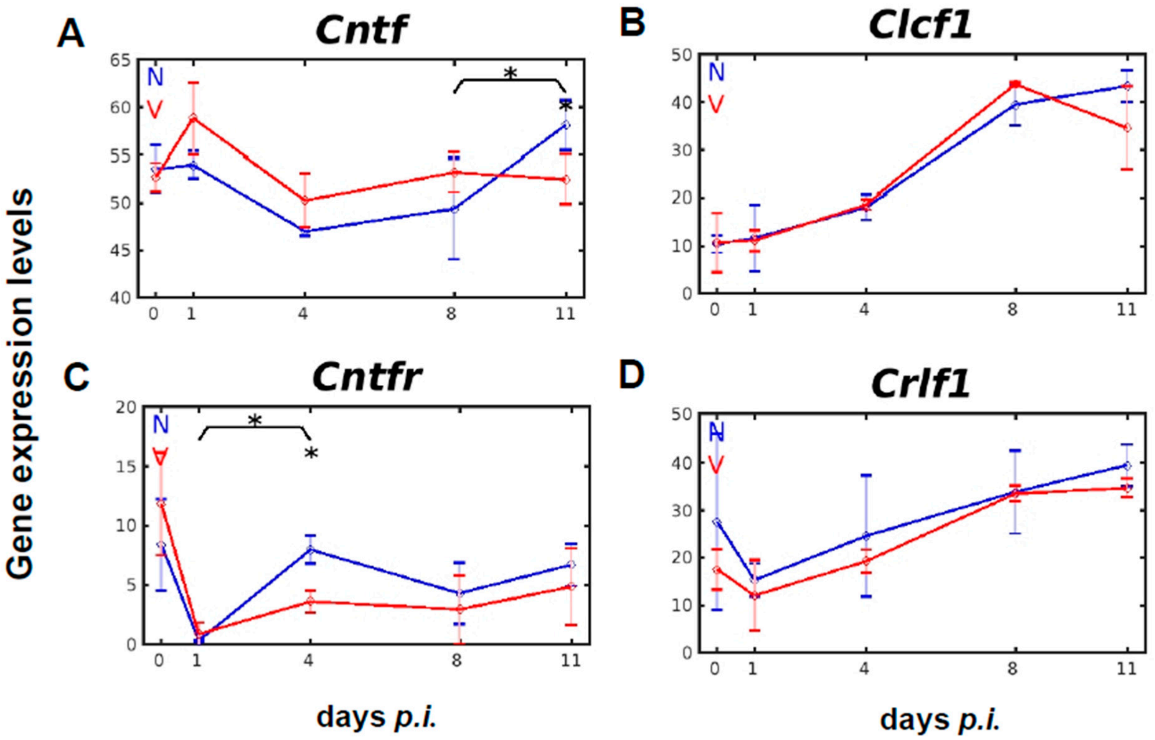

Figure S4. Expression trajectories of genes encoding different APPs in the liver in response to blood-stage malaria and vaccination. (A) *Cntf*. (B) *Clcf1*. (C) *Cntfr*. (D) *Crlf1*. RNA was isolated from individual livers taken from vaccination-protected (V, red) and non-vaccinated (N, blue) mice on days 0, 1, 4, 8, and 11 *p.i.* during primary infections with *P. chabaudi* blood-stage malaria. Gene expression levels are plotted in linear scale as the mean of three microarrays +/- SD. The '*' symbol over the sampling points or interval lines between two sampling points indicates a *p*-value<0.05 statistically significant difference at a given sampling point or two corresponding intervals between vaccinated and non-vaccinated mice. Numerical *p*-values are provided in Table S12. $n_N = 3$ and $n_V = 3$ are the total number of non-vaccinated and vaccinated mice, respectively, at each time point.
